# Comparison of Two Different Methods to Evaluate Ankle Syndesmosis on Lateral Ankle Radiographs

**DOI:** 10.7759/cureus.51070

**Published:** 2023-12-25

**Authors:** Abdelfatah M Elsenosy, Islam H Mansy, Eslam Hassan, Senthil Muthian

**Affiliations:** 1 Trauma and Orthopaedics, University Hospital Dorset, Poole, GBR; 2 General Surgery and Surgical Oncology, Maadi Armed Forces Medical Complex, Cairo, EGY; 3 Trauma and Orthopaedics, Poole General Hospital, Poole, GBR

**Keywords:** anterior tibiofibular ratio, anteroposterior tibiofibular ratio, radiographs, lateral, ankle syndesmosis

## Abstract

Background

Ankle sprains and fractures rank among the most commonly occurring musculoskeletal injuries and hold significant relevance in current medical practice. Accurate information regarding these injuries is crucial for their effective treatment. This study aimed to explore the viability of utilizing ankle lateral radiographs to evaluate syndesmosis in both emergency settings and operating theatres.

Methods

This randomized retrospective clinical study involved the analysis of 150 ankle lateral radiographs (54 males and 96 females) from patients who presented at our emergency department with suspected ankle injuries. Two authors jointly examined these radiographs and reached a consensus. The anterior tibiofibular (ATF) ratio and anterior-posterior tibiofibular (APTF) ratio were computed. Patients requiring syndesmotic fixation were classified as having experienced a genuine syndesmotic injury. Participants were randomly divided into two equal groups: Group I (normal group) without fractures and Group II (abnormal group) with fractures. Comprehensive patient data, including medical history and clinical examinations, were recorded.

Results

Gender distribution within the studied population consisted of 54.67% males (n=41) and 45.33% females (n=34) in the abnormal group, while the normal group comprised 37.33% males (n=28) and 62.67% females (n=47). Both APTFR and ATFR methods were found to be inconclusive and unreliable for syndesmosis assessment in ankles. The sensitivity of APTFR stood at 21.33%, with a specificity of 86.67%, a positive predictive value (PPV) of 61.5%, and a negative predictive value (NPV) of 52.4%. Meanwhile, the sensitivity of ATFR was 32%, with a specificity of 80%, a PPV of 61.5%, and an NPV of 54.1%.

Conclusions

Both techniques demonstrated low sensitivity when ankle fractures were present, indicating their unsuitability for routine clinical diagnosis of syndesmotic disruption via lateral ankle radiographs.

## Introduction

Sprains and fractures of the ankle are among the most prevalent musculoskeletal injuries, as well as the most recent and pertinent [1]. This information is crucial when treating such injuries [1,2]. The radiographic examination of the ankle has been extensively studied throughout the last 20 to 30 years. Measurements for radiography have been created to record the anatomy of the ankle; Pettrone and associates identified significant predictive characteristics after looking at 146 misplaced ankle fractures [3]. The researchers continued to employ the objective measurements utilized in their study in both contemporary clinical practice and research [3].

However, our standard diagnostic imaging often inadequately represents certain ankle components. When radiography is deemed necessary for an ankle, the typical procedure involves obtaining anteroposterior (AP), lateral, and mortise views [4]. A critical analysis of these images highlights the syndesmosis as the most clinically significant soft tissue element of the ankle. The syndesmosis refers to a fibrous connection established by ligaments or a robust membrane that links two adjacent bones. Specifically, this description encompasses the distal tibiofibular syndesmosis, a syndesmotic joint comprising two bones and four ligaments [5].

Four ligaments make up the syndesmosis: the inferior transverse tibiofibular ligament, the interosseus tibiofibular ligament/membrane, the anterior inferior tibiofibular ligament, and the posterior inferior tibiofibular ligament [6]. The syndesmosis connects the distal tibia and fibula, and damage to it can cause serious acute and long-term morbidity [6]. According to estimates, a syndesmotic injury coexists with 13% of all ankle fractures and 20% of fractures that require surgical treatment [7]. Cedell found that only 1% to 10% of ankle ligament injuries (medial collateral, lateral collateral, and interosseous ligaments) were syndesmotic sprains [8]. Some people think that in athletes, the incidence could reach 40%. The most widely used clinical diagnostics for identifying syndesmosis injuries are listed in these studies. However, none of these examinations have a strong ability to predict acute syndesmosis disruption [9].

The AP and mortise views have traditionally been used to characterise radiographic characteristics to identify a syndesmotic lesion. A mortise view should show that the superior joint space is within 2 mm medially of its breadth laterally, and the medial joint space should be less than 4 mm [10]. More than 10 mm should be the tibiofibular overlap on the AP view, and less than 5 mm should separate the tibia's incisural surface from the medial wall of the fibula [11].

On the other hand, recent research indicates that assessing the syndesmosis on static AP and mortise views may not be sufficient to identify whether a syndesmotic injury is present. According to certain suggestions, the diagnostic criteria now in use for syndesmotic injuries are not very useful, and new criteria ought to be created [6,12]. The diagnosis of syndesmotic injuries can be ensured more accurately by using computed tomography (CT), magnetic resonance imaging (MRI), or ankle arthroscopy instead of routine radiography. Nevertheless, none of these methods is clinically or financially feasible [13,14]. Increasing the orthogonal parameters on the lateral radiograph to those in the AP and mortise views could be one way to solve this problem and make them more effective [15].

This study's main objective was to determine the possibility of using ankle lateral radiographs to assess the syndesmosis emergency settings and operating theatres.

## Materials and methods

This randomized retrospective clinical study was conducted on 150 ankle lateral radiographs of patients who attended our emergency department at University Hospital Dorset, UK.

The study population consisted of 75 patients in each group. The abnormal group, which included individuals with fractures, was further divided into three categories based on the severity of the fracture: 56% (n=42) had isolated malleolar fractures, 28% (n=21) had bimalleolar fractures, and 16% (n=12) had trimalleolar fractures. On the other hand, the normal group consisted of individuals without any fractures.

The anterior tibiofibular (ATF) ratio and anterior-posterior tibiofibular (APTF) ratio were calculated. Any patient who needed syndesmotic fixation was considered as having sustained a true syndesmotic injury, in which the outpatient clinic notes were analyzed.

The radiographs included in this study satisfied all of the following requirements: the patient had to be 21 years of age or older, there had to be no known ankle joint disease or condition, the radiographs had to show a true lateral ankle view with the talar domes superimposed, and the X-ray had to be taken by a senior radiologic technician.

The radiographs included in this study satisfied all of the following requirements: the patient had to be 21 years of age or older, there had to be no known ankle joint disease or condition, the radiographs had to show a true lateral ankle view with the talar domes superimposed, and the X-ray had to be taken by a senior radiologic technician. The following types of radiographs were not included: poorly taken radiographs with blurry films or artefacts, patients who had previously undergone ankle surgery, patients who had previously experienced ankle trauma, and pregnant patients.

Randomization

A computer-generated list of random numbers, which was securely encased in an opaque envelope, was utilized to randomly allocate the participants into two equal groups on a 1:1 scale. In the abnormal group, it was Males (34.67%, n=26) and Females (65.33%, n=49). In the normal group, Males (37.33%, n=28) and females (62.67%, n=47).

All data about the patients were collected as full history taking and clinical examination. The radiographs were retrospectively gathered through the picture archiving and communication system (PACS) system at our trust. The lines and measures were done by three independent observers who measured the anterior tibiofibular interval (ATFI) and the posterior tibiofibular interval (PTFI) of the lateral ankle radiographs to ensure accuracy and avoid bias in the results. Two measurements were recorded: ATFI, which corresponds to the distance between the anterior cortex of the fibula and the anterior cortex of the tibia, and PTFI, which is described as the posterior cortex of the tibia to the posterior cortex of the fibula [16]. Measurements were recorded 1 cm above the tibial plafond's centre [17,18].

Instruction 1

**Figure 1:**
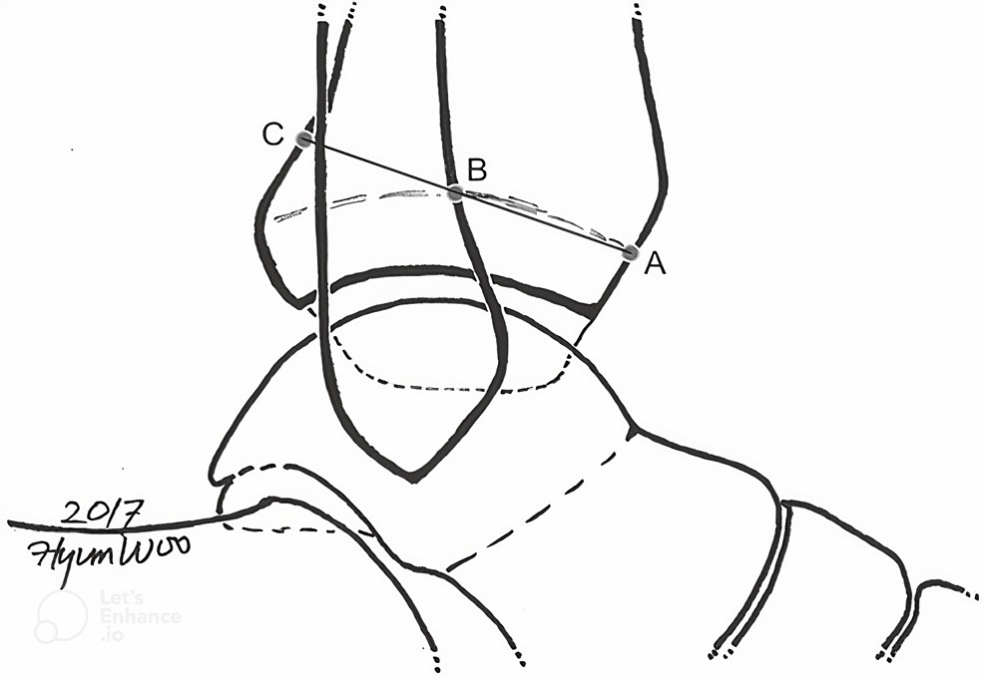
Grenier's method: anteroposterior tibiofibular (APTF) ratio A: anterior cortex of the tibia at the level of the physeal scar, B: the intersection of the anterior cortex of the fibula and the tibial physeal scar, C: the intersection of the line crossing A and B and the posterior cortex of the tibia Anteroposterior tibiofibular ratio = AB/BC Source: [19] (This is an open-access article distributed under the terms of the Creative Commons Attribution Non-Commercial License (http://creativecommons.org/licenses/by-nc/4.0)

A radiopaque transverse fusion line that represented the physeal scar was visible in the lateral view of the X-ray. (AB) measured the distance from the tibia's anterior cortex at the physeal scar level to the intersection of the tibial physeal scar and the anterior cortex of the fibula. (BC) continued as the line crossed A and B and was measured to the tibia's posterior cortex (Figure 1).

Instruction 2

**Figure 2:**
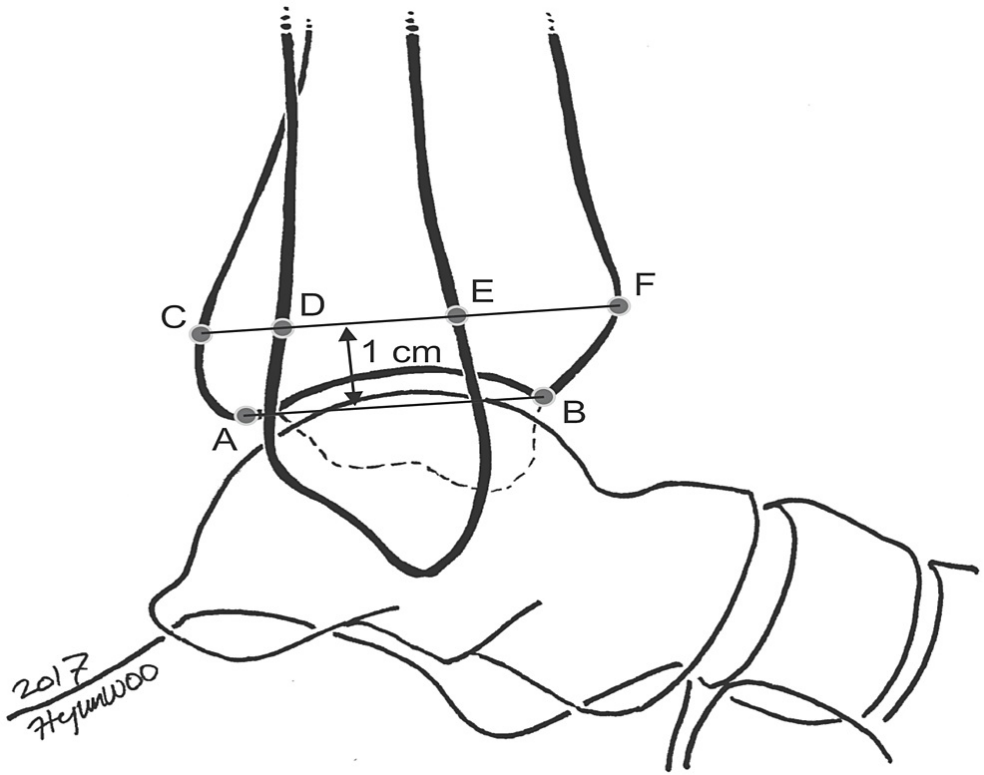
Croft method: the anterior tibiofibular ratio is defined as the ratio of the tibial width to the anterior tibiofibular interval The line segment A–B shows the tibial plafond, C–D shows the posterior tibiofibular interval, D–E shows the fibular width, E–F shows the anterior tibiofibular interval, and C–F shows the tibial width. All measurements were made 1 cm above the midpoint of the tibial plafond. Source: [19] (This is an open-access article distributed under the terms of the Creative Commons Attribution Non-Commercial License (http://creativecommons.org/licenses/by-nc/4.0)

**Figure 3:**
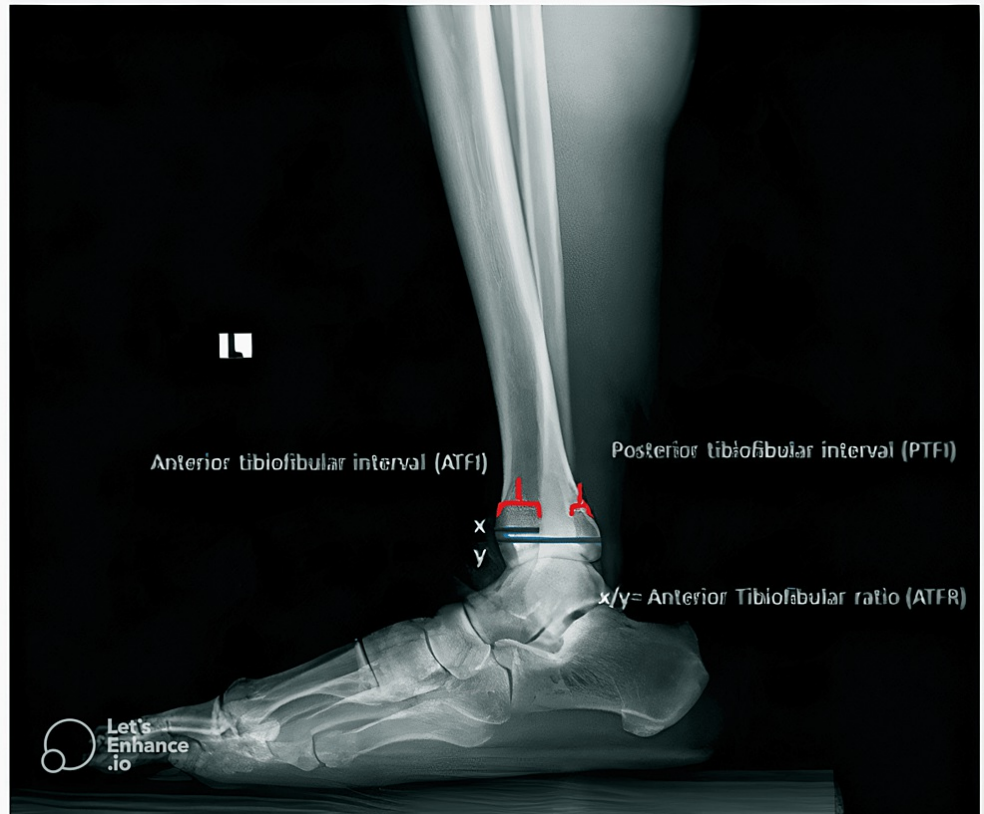
Lateral radiograph measurements

In the lateral view X-ray, a midpoint (AB) to document the rest of the measurements, 1 cm was taken from the midpoint of the tibial plafond, (CF) the tibial width - y line was measured and the (EF) anterior tibiofibular interval (ATFI) described as the distance between the anterior cortex of the tibia and the anterior cortex of the fibula - x line was measured (Figure 2, Figure 3).

Statistical analysis

SPSS (Statistical Package for the Social Sciences) v28 (IBM Corp., Armonk, NY) was utilized for the statistical analysis (IBM Inc., Armonk, NY, USA). The unpaired student's t-test was utilized to contrast the two groups based on quantitative data that were reported as mean and standard deviation (SD). When appropriate, Fisher's exact test or Chi-square test was utilized to analyse the frequency and percentage (%) of the qualitative variables. For statistical significance, a two-tailed P value less than 0.05 was used. Evaluation of diagnostic performance was performed by evaluating the receiver operating characteristic curve (ROC-curve) analysis, positive predictive value (PPV), negative predictive value (NPV), specificity, and sensitivity of the diagnostic. The area under the curve (AUC) evaluated the overall test performance.

## Results

**Figure 4:**
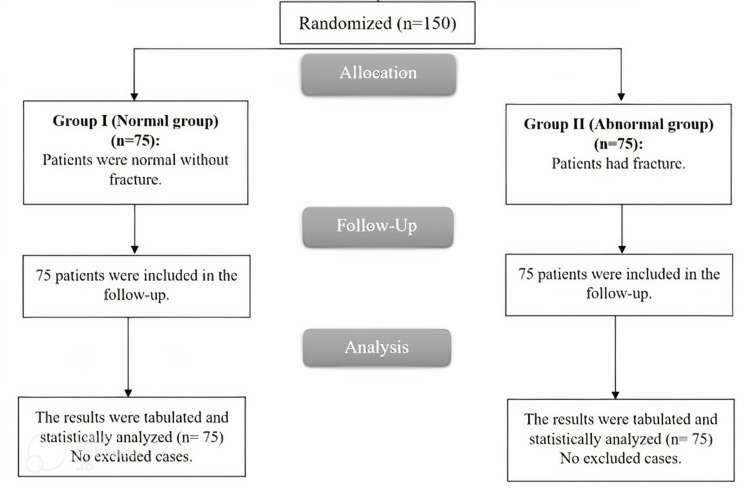
CONSORT flowchart of the enrolled patients

For this investigation, 150 patients were divided into two groups at random (75 patients in each). Every patient that was assigned was tracked down and statistically examined (Figure 4).

Table 1 shows that age was significantly greater in the abnormal group in contrast to the normal group (P=0.001), sex and side were insignificantly different between both groups. Regarding the severity of the fracture in the abnormal group, 42 (56%) patients had isolated malleolar fractures, 21 (28%) patients had bimalleolar fractures and 12 (16%) patients had trimalleolar fractures. All patients in the normal group had no fracture. The severity of fracture was significantly different between both groups (P<0.001), as all patients in the normal group had no fractures, unlike all patients in the abnormal group.

**Table 1:** Baseline characteristics of the studied groups Data displayed as mean ± standard deviation (SD) or frequency (%), *: statistically significant as P value <0.05.

		Normal group (n=75)	Abnormal group (n=75)	P value
Age (years)		44.0 ± 25.85	57.4 ± 20.68	0.001*
Sex	Male	28 (37.33%)	26 (34.67%)	0.734
	Female	47 (62.67%)	49 (65.33%)	
Side	Right	44 (58.67%)	41 (54.67%)	0.621
	Left	31 (41.33%)	34 (45.33%)	
Severity of fracture	No fracture	75 (100%)	0 (0%)	< 0.001*
	Isolated malleolar	0 (0%)	42 (56%)	
	Bimalleolar	0 (0%)	21 (28%)	
	Trimalleolar	0 (0%)	12 (16%)	

On the lateral view X-ray, BC by the first, second and third observers were significantly greater in the abnormal group in contrast to the normal group (P<0.05), meanwhile, AB by the first, second and third observers were insignificantly different between both groups (Table 2).

**Table 2:** Findings of the lateral view X-ray of the studied groups Data displayed as mean ± standard deviation (SD), *: statistically significant as P value <0.05.

		Normal group (n=75)	Abnormal group (n=75)	P value
AB	1^st^ observer	15.4 ± 4.23	16.6 ± 7.35	0.245
	2^nd^ observer	15.0 ± 4.86	16.4 ± 6.95	0.164
	3^rd^ observer	14.96 ± 4.29	16.4 ± 7.31	0.135
BC	1^st^ observer	24.2 ± 5.27	27.04 ± 6.8	0.005*
	2^nd^ observer	24.6 ± 5.13	27.01 ± 6.52	0.012*
	3^rd^ observer	24.2 ± 4.93	27.2 ± 6.32	0.001*

Table 3 shows that there were insignificant differences between the studied groups concerning the Grenier method (APTF ratio) and Croft method (ATFR) by the first, second and third observers.

**Table 3:** Grenier method (APTF ratio) and Croft method (ATFR) of the studied groups Data presented as median (IQR) or mean ± SD, IQR: interquartile range, APTF: anteroposterior tibiofibular, ATFR: anterior tibiofibular ratio

		Normal group (n=75)	Abnormal group (n=75)	P value
APTF ratio	1^st^ observer	0.64 (0.47-0.89)	0.58 (0.42-0.77)	0.169
	2^nd^ observer	0.59 (0.46 -0.81)	0.58 (0.45-0.75)	0.565
	3^rd^ observer	0.63 (0.48-0.88)	0.58 (0.41-0.76)	0.117
ATFR	1^st^ observer	3.04 ± 0.94	3.4 ± 1.46	0.071
	2^nd^ observer	3.2 ± 1.11	3.1 ± 1.26	0.919
	3^rd^ observer	0.4 ± 0.09	0.3 ± 0.13	0.293

Y-line by the first, second and third observers were significantly greater in the abnormal group in contrast to the normal group (P<0.05), whereas the X-line by the first, second and third observers were insignificantly different between both groups (Table 4).

**Table 4:** X and Y lines of the studied groups Data displayed as mean ± SD, APTF: anteroposterior tibiofibular, *: statistically significant as P value <0.05

		Normal group (n=75)	Abnormal group (n=75)	P value
X-line	1^st^ observer	14.7 ± 4.24	15.9 ± 7.4	0.235
	2^nd^ observer	14.5 ± 5.05	16.02 ± 7.21	0.130
	3^rd^ observer	14.6 ± 4.26	15.6 ± 7.26	0.321
Y-line	1^st^ observer	41.5 ± 4.59	45.8 ± 6.49	<0.001*
	2^nd^ observer	41.2 ± 4.93	45.1 ± 6.7	<0.001*
	3^rd^ observer	41.3 ± 4.56	45.9 ± 6.54	<0.001*

In the abnormal group, 44 (58.67%) patients had non-operative fracture management and 31 (41.33%) patients had operative management. Among the studied patients, 5 (6.67%) underwent posterior malleolus fixation (Table 5).

**Table 5:** Fracture management and posterior malleolus fixation of the studied patients in the abnormal group Data presented as mean ± SD, ATFR: anterior tibiofibular ratio

		Abnormal group (n=75)
Fracture management	Non-operative	44 (58.67%)
	Operative	31 (41.33%)
Posterior malleolus fixation	Yes	5 (6.67%)
	No	70 (93.33%)

Table 6 shows that 74 (98.67%) patients in the normal group and 68 (90.67%) patients in the abnormal group had no screws for syndesmotic fixation, only 1 (1.33%) patient in the abnormal group had syndesmotic rope (tight rope) and 1 (1.33%) patient in the normal group and 6 (8%) patients in the abnormal group had syndesmotic screws. There was an insignificant difference between both groups regarding the syndesmotic fixation.

**Table 6:** Syndesmotic fixation of the studied groups Data presented as frequency (%), *: statistically significant as P value <0.05

		Normal group (n=75)	Abnormal group (n=75)	P value
Syndesmotic fixation	No screw	74 (98.67%)	68 (90.67%)	0.089
	Syndesmotic rope	0 (0%)	1 (1.33%)	
	Syndesmotic screws	1 (1.33%)	6 (8%)	

Table 7 shows that both APTF and ATFR were insignificant and unreliable methods for the evaluation of ankle syndesmosis. The sensitivity of APTF was 21.33%, the specificity was 86.67%, the PPV was 61.5% and the NPV was 52.4%. The sensitivity of ATFR was 32%, the specificity was 80%, the PPV was 61.5% and the NPV was 54.1%.

**Table 7:** Diagnostic accuracy of APTF and ATFR for evaluation of ankle syndesmosis APTF: anteroposterior tibiofibular, ATFR: anterior tibiofibular ratio, PPV: positive predictive value, NPV: negative predictive value, AUC: area under the curve

	Criterion	Sensitivity	95% CI	Specificity	95% CI	PPV	NPV	AUC	P
APTF	≤0.380	21.33	12.7 - 32.3	86.67	76.8 - 93.4	61.5	52.4	0.565	0.168
ATFR	>3.689	32	21.7 - 43.8	80	69.2 - 88.4	61.5	54.1	0.559	0.218

## Discussion

The most important ligamentous complex in the ankle is the syndesmosis [20]. Instability, discomfort and arthrosis can result from disturbance [21]. Malreduced syndesmotic injuries have been associated with inferior functional results as measured by the Olerud/Molander questionnaire, which is specific to the ankle, and the short-form musculoskeletal assessment, which evaluates general health [22,23]. Nielson and colleagues [24] and Hermans and colleagues [25] compared MRI findings to conventional radiography assessments for ankle fractures. The authors discovered that there was no correlation between MRI and radiography data for syndesmosis damage [24].

Numerous instances of sagittal displacement of the fibula in relation to the tibia have been reported in the literature as indicative of a syndesmotic injury [26-28]; nevertheless, only earlier research has made an effort to identify precise diagnostic criteria from a lateral perspective [17,27]. Assessment of the syndesmosis on the AP and mortise views of the ankle was advised using radiographic measures. Nonetheless, Beumer et al. thought that these measures were not the best for evaluating ankle syndesmosis [28]. In fact, CT scans and MRIs are thought to be better options than conventional radiographs for accurately evaluating syndesmosis [29]. In contrast, certain surgeons may not typically have access to intraoperative CT scan evaluation [15].

We found that both APTF and ATFR were deemed unreliable methods for assessing ankle syndesmosis through these results.

Grenier et al. revealed a novel way to quantify ankle syndesmosis radiographically using the true lateral view ankle radiograph, which may be utilized to assess syndesmosis intraoperatively: the anteroposterior tibiofibular ratio (APTF) [27]. The APTF ratio ranged from 0.63 to 1.31, with an average of 0.94 ± 0.13. The authors concluded that this ratio represents a novel, trustworthy technique for radiographically assessing the distal tibiofibular joint structure. The results of this investigation indicated an APTF ratio of 0.90 ± 0.08 with a range of 0.75 - 1.2. Meanwhile, Croft and colleagues further assessed lateral ankle radiographs using the ATFI, PTFI, FW and tibial width (TW) measures [15]. These measures were used to analyze the tibia and fibula relationship using four ratios: PTFI: TW, ATFI: TW, PTFI: (PTFI + FW) and ATFI: (ATFI + FW). The ATFR (ATFI: TW) ratio has a higher intraclass correlation coefficient among the four ratios, according to the authors' findings. The ATFR, which corresponds to 39% ± 9% of the tibia, should be located anterior to the anterior fibular cortex, according to their findings. Because of this, the authors suggested using the ATFR in addition to other metrics to assess syndesmotic disruptions. The authors found that, when measured 1 cm above the tibial plafond centre, the ATFR of 0.41 ± 0.07, with a range of 0.26 - 0.68, or 41% ± 7%of the tibia, is anterior to the anterior fibular cortex. Iturriaga et al. conducted a pilot investigation on a total of 40 lateral ankle radiographs and found that, in order to assess ankle syndesmosis, APTF and ATFR are two radiographic parameters on the lateral ankle that should be added to other imaging and diagnostic studies [30]. Suh et al. examined 34 ankle fracture cases using a postoperative ankle radiograph following screw fixation for a concomitant syndesmosis injury [19]. On every AP and mortise radiograph, the radiographic parameters tibiofibular overlap (TFO) and tibiofibular clear space (TFCS) were assessed. On the genuine lateral radiographs, the following five radiographic characteristics were measured: the anterior tibiofibular ratio (ATFR), posterior tibiofibular ratio (PTFR) and anteroposterior tibiofibular ratio (APTF).

The fact that our study is limited to radiographic examinations of healthy individuals is one of its advantages. This was left out of earlier research.

The inherent limitations of a retrospective approach, such as potential selection bias, apply to our work. The detection and treatment of the proper pathology will be facilitated by ensuring adequate and high-quality radiography.

## Conclusions

Based on the results, it seems that the two techniques we are referring to (Croft and Grenier) have a low rate of sensitivity in the presence of ankle fractures and hence cannot be used in routine clinical practice for the diagnosis of syndesmotic disruption on lateral ankle radiographs. It is important to note that the two methods were described in patients without ankle fractures, and further randomized larger cohorts are necessary to validate the findings.
